# Can Identity Buffer Against the Detrimental Effects of Threat? The Case of the Qatar Blockade

**DOI:** 10.3389/fpsyg.2022.750471

**Published:** 2022-03-30

**Authors:** Azzam Amin, Jasper Van Assche, Mohamed Abdelrahman, Darragh McCashin, Duaa Al-Adwan, Youssef Hasan

**Affiliations:** ^1^Doha Institute for Graduate Studies, Doha, Qatar; ^2^Department of Developmental, Personality and Social Psychology, Ghent University, Ghent, Belgium; ^3^Center for Social and Cultural Psychology, Université Libre de Bruxelles, Bruxelles, Belgium; ^4^Psychology Program, Department of Social Sciences, College of Arts and Sciences, Qatar University, Doha, Qatar

**Keywords:** perceived threat, national identity, self-esteem, well-being, Qatar blockade

## Abstract

In 2017, the blockade of Qatar Gulf states caused a plethora of effects on the country. This paper sought to examine the resulting threat effects of this blockade in terms of lowered self-esteem and well-being, and the potential buffering effects of an overarching identity. Using self-report questionnaire data from Qatari secondary school students (*N* = 1,410), multiple moderated mediation models investigated the predictive effects of youngsters’ perceived threat, *via* self-esteem, on their well-being, and the mitigating roles herein of, respectively, national, Gulf region, and Arab identity. Perceived threat was indeed related to lower well-being *via* lower self-esteem, and this relationship was equally strong for those low and high in social identity. In terms of the three facets of identity, the overarching Gulf identity seems the most predictive, and it even (marginally significantly) buffers the negative relationship between threat and reduced self-esteem.

## Introduction

In 1981, six Arab countries (the Kingdom of Saudi Arabia, Qatar, the Kingdom of Bahrain, the United Arab Emirates, Kuwait, and Oman) signed an agreement to establish the Gulf Cooperation Council (GCC). This charter contributed significantly to the stability in the region., the GCC region has even been considered the most stable entity of the Middle East region (Bianco and Stansfield, 2018). The fundamental principles of constructing this entity were to promote economic, financial, and cultural cooperation, to enhance social ties between people, and to foster political stability and security within the region (Nakhleh, 1986).

From a social identity perspective, the charter also helped to facilitate an integrated entity among the Gulf states and its citizens. It enabled people in all Gulf states to develop a shared identity known as “Khaleeji’ identity” (Al-Misned, 2016). Historically, this overarching Gulf identity had been formed long before the separate Gulf states, and their according national identities, emerged (Allam and Karolak, 2020). In relation to social ties, intermarriage across GCC countries is common. As a result, the existence of extended families across these six countries produced similarities in many aspects of life spanning culture, identity, music, and poetry. In addition, GCC citizens have travel privileges to facilitate free movement between member states without visa requirements (Alshihaby, 2015). Therefore, these factors indicate that GCC citizens perceived themselves as relatively united and with a common identity sharing many key characteristics (Al-Khouri, 2010).

### The 2017 Blockade of Qatar

Aside from the geopolitical importance of the landscape, the Gulf region is undergoing considerable political and social transformations caused by several key trends in recent times, including the Arab Spring, economic transitions, and shifting demographics. The state of Qatar—a small peninsula within the Arabian Gulf—is a traditional Muslim collectivist society with established gender segregation norms (e.g., separate boys and girls schools; Bahry and Marr, 2005); but with state commitments to harmoniously modernize the country with ambitious development strategies at national and international levels, such as the hosting of the FIFA World Cup 2022. High levels of social security, extensive public and private investments, openness to globalization and rapid industrialization have typified the nature of Qatar’s development in recent decades (Dogan Akkas and Camden, 2020). Such developments have also led to the emergence of regional competitiveness, with Qatar and the United Arab Emirates (UAE) regularly competing with each other to promote their active contributions to international society (Ennis, 2018). As a result, it has been suggested that the success of Qatar in different fields such as sport can foster jealousy (Gulf Times, 2017).

Although the GCC entity has maintained stability and cohesion since its inception, the political relationships have encountered some tensions among the allied countries. For example, there was a sovereignty dispute between Bahrain, Qatar and the Hawar Islands in 1936, which was peacefully resolved in 2001 *via* the International Court of Justice (Wiegand, 2012). Similarly, political tension resulted in a border dispute between Qatar and Saudi Arabia in 1992 and 1994 (Okruhlik and Conge, 1999). Nonetheless, the GCC overcame these disputes and maintained the strong ties among the Gulf states.

However, on 5th June 2017, to much regional and international shock, the Gulf States of the Kingdom of Saudi Arabia, the Kingdom of Bahrain, the United Arab Emirates; and Egypt all severed diplomatic relations with Qatar. Given the cooperation of the Gulf region in the past, this blockade was regarded as unprecedented; and brought with it a cascade of effects across many levels of society. Sophisticated cyberattacks on Qatari state media implicated Qatari stakeholders across a range of politically sensitive allegations. Following this, a 13-point list of maximalist demands were given to Qatar (Ulrichsen, 2020)—itself underpinned by a narrative of Qatari involvement in terrorist activity and corruption (Milton-Edwards, 2020). For example, the demands included: closing of Qatari-run media such as Al Jazeera, ceasing military cooperation with Turkey, severing alleged ties with terrorist organizations and the hosting of their representatives within Qatar, and paying compensation to the blockading Gulf states.

Despite a willingness to find a resolution, Qatar did not wish to accept the accusations nor the demands of the blockade. Owing to considerable dependence on importations of key goods and services, in addition to the social and familial interconnectedness of Gulf states, immeasurable challenges emerged for the Qatari population. The Qatari National Human Rights Committee (QNHRC) in 2017 reported that the blockade had instilled a sense of fear due to the fragmentation of families (due to border closures), created risks of adverse psychological outcomes, and caused irreparable damage between once-intertwined Gulf cultures and societies. Undoubtedly, the feelings of threat accompanying the blockade may pose a serious challenge to the self-esteem and the well-being of Qatari people. Nonetheless, these psychological consequences of intergroup conflicts in the Gulf region remain poorly understood within the literature. The particular characteristics of this blockade present a unique opportunity to understand intergroup conflict within an understudied region.

### The Detrimental Effects of Threat

Research on (perceived) intergroup conflict has typically focused on effects relating to stressors that may exacerbate conflict, physicality, territory, power; in addition to restrictions for civil liberties and human rights (Carriere et al., 2020). For instance, previous studies on the European continent have indicated that threat is related to greater levels of prejudice [see e.g., Van Assche et al. (2018) for samples from Netherlands and Germany] and to lower levels of well-being [see Schmid and Muldoon (2015) for a Northern Irish sample]. In the latter context, this detrimental effect of threat was only for those who had prior experience with the particular political conflict or with the co-occurring violence. Yet, there is a distinct lack of research outside these so-called WEIRD (Western, Educated, Industrialized, Rich, and Democratic) contexts, and on the potential role of perceived threat for well-being and self-esteem (a well-known proxy of well-being) in young adults where such individuals do not have prior experience of conflicts, as is the case in the Qatari blockade.

The distinctive factors within the Qatari blockade are difficult to situate within the current perceived intergroup threat literature. This is due to a number of factors, chiefly the absence of violence, and the uniquely impactful role of social media in communicating some of the psychological effects of the blockade in young adults—living in an increasingly globalized Qatar—who have no direct experience of political conflict or violence (Alkaabi and Soliman, 2017; El-Masri et al., 2020). Moreover, over 50,000 citizens from the Kingdom of Saudi Arabia, the Kingdom of Bahrain, and the United Arab Emirates lived in Qatar prior to the blockade, and significant overlapping familial, sporting, commercial and political ties existed across the Gulf (Zahlan, 2016).

The largely unforeseen and immediate severing of these ties has never been experienced by the so-called generation Z of Qatari citizens (born from 1996 onward). Given the overlapping ties held by young Qatari adults across Gulf groups, it is unclear how the functionality of the perceived threats from the blockade would potentially affect individual self-esteem and well-being, depending on the commonality Qataris feel with other Gulf state citizens. It should be noted that perceived threats of any kinds may not adversely impact well-being in all contexts. There is a body of research that has demonstrated the important positive relationship between one’s social identity and perceived threats.

### Social Identity as a Potential Buffer

Whereas the threat by the blockade can have a negative impact on Qatari’s self-esteem and well-being, they can find solace in their social identity. In particular, this paper adopts a theoretical background initially framed by social identity theory (Tajfel et al., 1979) which posits that individuals’ sense of identity is based on their group membership(s), and can thus heavily influence other key psychological factors (including self-esteem, group pride, and even well-being). Social identity theory places the origins of social identity within the domains of both cognitive and motivational factors—these can influence group members to support or detach from their group. Indeed, one of the key contributions of social identity theory is the replicated finding of ingroup favoritism across different conditions (Brewer, 1979).

Of course, much depends on how broadly one defines one’s ingroup to be. To the extent that one identifies as Qatari, this national identity might be related to stronger preference for Qatar over other Gulf countries (and their citizens). Nevertheless, such national identity and ingroup favoritism has been shown to elevate one’s self-esteem (Rubin and Hewstone, 1998). To the extent that one identifies with the overarching category of Gulf states (i.e., “Khaleeji”), this superordinate common ingroup identity (e.g., Dovidio and Gaertner, 2000) might be related to a more broad-minded perspective on the intergroup tensions following the blockade. As such, this overarching identity might not only be positively related to self-esteem and well-being, it might even *buffer* against the detrimental effects of perceived threat. Exactly because this supranational identity incorporates a strong sense of connection with the other Gulf states, i.e., the very perpetrators imposing the blockade and potentially causing the feelings of threat, we hypothesize that this level of identification has the strongest potential to buffer against threat effects. Finally, to the extent that one identifies with the even larger category of Arabs globally, this social identity facet might relate positively to self-esteem and well-being, but its potential buffering effect on the impact of post-blockade threat perceptions might be limited, given the broadness of the identification that spans much wider than the conflict area *per se*.

To sum up, in the context of social identity theory, we put forward that some individuals may more readily identify with their perceived ingroup in an attempt to cope with the stressors originating from perceived outgroup threats (Haslam et al., 2005). The unique Qatari situation of contact between the sub-groups making up a nation (or set of nations) could lead to different levels of identification (national, supra-regional, and ethno-cultural/religious), which, can be a source of consolation in the face of the potentially detrimental impact of threat on self-esteem and well-being (Gaertner et al., 1996; Dovidio and Gaertner, 2000).

### The Current Study

The current study was set up against the backdrop of the Qatari blockade. We specifically focused on Qatari youth, as previous research demonstrated that mental health problems in Qatari young adults are comparatively frequent (Al-Attiyah and Nasser, 2016; Schoenbach et al., 2018). Other data from undergraduate samples reported higher self-rated levels of mental health and well-being (Abdel-Khalek, 2013). However, a recent cross-cultural telephone survey of non-migrant Qataris and migrants found that the former had lower levels of depression that was comparable to Western epidemiology (between 4.2% and 6.6%; see Khaled, 2019; Khaled and Gray, 2019). Within Qatari primary healthcare, it is estimated that approximately one-quarter of attendees had at least one psychiatric diagnosis (Ghuloum et al., 2011; Bener et al., 2015). However, much of this data is using smaller samples from college-aged participants or older adults in timeframes that predated the Qatari blockade (Ciftci et al., 2013; Zolezzi et al., 2017).

To date, there exists no high-quality dataset regarding the well-being, self-esteem, identity or perception of threat among young citizens in Qatar, and no research regarding the effects of the blockade on these psychological variables. Nonetheless, the *National Mental Health Strategy for Qatar, Changing Minds, Changing Lives 2013–18* has identified a need for a population health approach using an integrated system of care, with mental health and well-being named as a priority area. This study aims to fill this gap by examining a model where threat and identity additively and interactively predict well-being *via* self-esteem. In other words, we propose a moderated mediation model with threat as predictor, well-being as outcome, self-esteem as mediator, and social identity as moderator. Indeed, we first predict that threat will be negatively related to well-being *via* lower levels of self-esteem (mediation hypothesis). Secondly, anchored in the Common Ingroup Identity hypothesis within Social Identity Theory, we hypothesize that national (Qatari), supra-regional (Khaleeji/Gulf), and ethno-cultural/religious (Arab) identities will be positively related to self-esteem and well-being, and the supra-regional identity in particular might exert a buffering effect on the negative threat-self-esteem association (moderation hypothesis).

## Materials and Methods

### Sample Size and Participants

According to the annual statistics of education in the State of Qatar (2018), the total number of secondary schools is 62 (33 are secondary schools for boys), and the total number of Qatari students in the secondary schools is 13,946 (7,305 are females). Given that there are eight municipalities in Qatar, the authors listed the secondary schools located in each municipality and randomly selected the schools. The results were a total of twenty-six schools (13 of which were for boys)^1^. Following that, the targeted number of the participants of each school was 60 students (20 participants per grades 10, 11, and 12 each).

Data were collected *via* a self-report paper questionnaire between November 2019 and February 2020. Eligible participants had to be Qatari citizens from public secondary schools in Qatar. A representative sample of 1,500 participants was recruited using convenience sampling, and 50 incomplete responses and 40 non-Qatari respondents were excluded. The final sample included 1,410 participants, of which 40% were males (*M*_*age*_ = 16.98, *SD* = 0.86).

After receiving a signed consent form from their parents, respondents were presented with a clear description of the study and they provided written informed assent prior to completing the survey. Participation was voluntary and respondents were asked to complete the questionnaire at the school under the supervision of the school teachers and the recruited research assistants for the study.

### Measures

#### Perceived Threat

The authors developed a 5-items scale to assess the extent to which Qatari citizens feel threat as a result of blockade. Examples of the items used were “I feel anxious when I think of the blockade crisis” and “I feel fearful when I think of the blockade crisis.” The participants were asked to rate their responses on a 7-point Likert scale ranges from 1 = strongly disagree to 7 = strongly agree. A CFA showed the suitability of the scale, where RMSEA = 0.022; SRMR = 0.006; CFI = 0.99; NFI = 0.99; GFI = 0.99; and TLI = 0.98. The internal consistency showed a good reliability (Cronbach’s α = 0.84).

#### Social Identity

To measure national (Qatari) identity, we selected four items from the Arabic version of the national identity scale developed by Al Rabaani (2017). Such a scale was developed and used for Omani secondary school students in the same education stage as their Qatari counterparts. Moreover, since Qatar and Oman have a shared Khaleeji culture, the authors decided to use the same scale with small adaptation where the word “Omani” was replaced with “Qatari.” Besides, the scale indicated a very good internal consistency in the Omani sample (α = 0.94). Participants were asked to rate the items on a 7-point Likert scale from 1 = strongly disagree to 7 = strongly agree (for example, “I am proud of being Qatari”). A confirmatory factor analysis (CFA) showed the suitability of the scale, where RMSEA = 0.001; SRMR = 0.001; CFI = 0.99; NFI = 0.99; GFI = 0.99; and TLI = 0.99. The internal consistency of the scale was very strong (Cronbach’s α = 0.88).

To measure supra-regional (Gulf) identity and ethno-cultural (Arab) identity, we used the same scale that was used to assess the national identity and replaced the word Qatar with Khaleeji and Arabi for the two scales. For example, “I am proud of being Khaleeji” and “I am proud of being Arabi.” A confirmatory factor analysis for each scale indicated adequate model fit, where the model fit indices for the Gulf identity scale were RMSEA = 0.088; SRMR = 0.014; CFI = 0.99; NFI = 0.99; GFI = 0.99; and TLI = 0.99 and those for the Arab identity were RMSEA = 0.098; SRMR = 0.021; CFI = 0.98; NFI = 0.98; GFI = 0.99; and TLI = 0.96. The Cronbach’s Alpha for the Gulf and Arab identity scales was 0.87 and 0.83, respectively.

#### Self-Esteem

A 10-item scale of Rosenberg (1965) was used. Items included questions such as “At times I think I am no good at all (*reverse coded*)” and “I take a positive attitude toward myself.” Each item was rated on a 7-point Likert scale ranging from 1 = strongly disagree to 7 = strongly agree. The authors used the Arabic version of Gradat (2006). A CFA was performed and showed an acceptable model fit of the scale, where RMSEA = 0.079; SRMR = 0.057; CFI = 0.90; NFI = 0.90; GFI = 0.95; and TLI = 0.86. The internal consistency was acceptable where Cronbach’s Alpha coefficient was 0.71.

#### Well-Being

We opted for a broad and general assessment of different facets of psychological well-being (for a similar approach, see Costabile et al., 2021). Relying on the work of Seligman (2011), we defined well-being in terms of the five “PERMA” pillars: Positive emotion, Engagement, Relationships, Meaning, and Accomplishment. A 15-item PERMA-Profiler was used. This scale was developed by Butler and Kern (2016). The scale was translated by a bilingual mental health specialist and back translated by a certified translator to avoid ambiguity of the items. Participants were asked to report their answers on a 10-point scale from 1 = strongly disagree to 10 = strongly agree. The items included “How often do you feel joyful” and “To what extent you have been feeling loved.” A CFA was carried out and showed an acceptable model fit of the scale, where RMSEA = 0.040; SRMR = 0.003; CFI = 0.97; NFI = 0.97; GFI = 0.98; and TLI = 0.97. The internal consistency of the scale was very strong (Cronbach’s α = 0.90).

### Statistical Analysis

To investigate our hypotheses, SPSS Version 26 software was used. Our data were theoretically nested (i.e., pupils were nested within schools). Therefore, we first investigated whether multilevel analyses were warranted. We estimated empty (intercept-only) models, which provide insight into the variances in our mediator and outcome at the individual and contextual levels. We also assessed the intraclass correlations (ICCs) to explore if there was substantial between-level variance in the scores of our mediator and outcome variable, which would warrant the use of multilevel modeling. Taking into account the higher-level structure did not significantly improve the goodness-of-fit statistics of each model (i.e., changes in −2 × log-likelihood were χ^2^ (1) = 1.86, *p* = 0.17 for self-esteem; and χ^2^ (1) = 0.46, *p* = 0.49 for well-being. Additionally, all ICC’s were very small (0.0056 for self-esteem and 0.0028 for well-being), indicating that only 0.56% of the variance in self-esteem and only 0.28% of the variance in well-being are due to differences at the school level. As such, multilevel analyses are not warranted.

Pearson correlation coefficients were calculated to explore the relationships among the study variables. To test the conditional indirect effects of threat on well-being *via* self-esteem at different levels of social identity, we conducted bootstrap analyses (1,000 bootstrap samples) using Hayes’ (2013) Process macro in which the association between the predictor (i.e., threat) and the mediator (i.e., self-esteem), as well as the associations between the predictor and the outcome variable (i.e., well-being) were moderated by social identity faces (i.e., Model 8; Hayes, 2013). In particular, we tested three such models, testing the separate buffering effects of national, supra-regional, and ethno-cultural identities, respectively (while controlling for the other two identity facets).

## Results

### Descriptive Statistics

Table 1 presents the means, standard deviations, and correlations between all study variables. At the outset, there were high overall levels of all three facets of social identity, and of self-esteem and well-being in the sample. The identity facets were all strongly positively interrelated, and they were all positively related to self-esteem and well-being. The mean score for perceived threat—a 7-point Likert scale—was considerably lower. Furthermore, there was no significant relationship between perceived threat and the identity facets, although threat was significantly negatively associated with self-esteem and well-being.

**TABLE 1:** Correlations among study variables.

Measure	*M*	*SD*	1	2	3	4	5	6
Perceived threat	2.91	1.52	–					
Qatari identity	6.87	0.53	0.02	–				
Gulf identity	6.40	0.95	−0.02	0.53***	–			
Arab identity	6.61	0.74	−0.04	0.60***	0.66***	–		
Self-esteem	4.95	0.80	−0.13***	0.27***	0.26***	0.29***	–	
Well-being	7.82	1.49	−0.13***	0.20***	0.25***	0.23***	0.49***	–

^a^p < 0.10; *p < 0.05; **p < 0.01; ***p < 0.001.

### Predicting Qatari Youth Well-Being

Table 2 portrays the results of the moderated mediation analyses. First, threat (negatively) and each identity facet (positively) independently predicted self-esteem. The interaction terms were not statistically significant, except for the small yet marginally significant interaction effect of threat and Gulf identity in the prediction of self-esteem. As predicted, simple slope analyses indicated that Gulf identity seemed to buffer the negative threat effects, in the sense that the threat effect was considerably lower for high Gulf identifiers than for low Gulf identifiers. Notably, the negative effect of threat, although small, was still significant among high Gulf identifiers, indicating that the buffering effect of this overarching social identity is rather limited. For Qatar and Arab identity, a similar buffering trend was found, but none of the slopes were significantly different between high and low identifiers.

**TABLE 2:** Unstandardized estimates and 95% confidence intervals of moderated mediation analyses on self-esteem (upper panel) and well-being (lower panel) for each facet of social identity separately.

	Moderator:								
	Qatar identity			Gulf identity			Arab Identity		
	Estimate	95% CI		Estimate	95% CI		Estimate	95% CI	
Outcome: self-esteem	b	LL	UL	b	LL	UL	B	LL	UL
Perceived threat	−0.07***	−0.09	−0.04	−0.07***	−0.09	−0.04	−0.07***	−0.09	−0.04
Social identity	0.23***	0.14	0.32	0.07**	0.02	0.13	0.15***	0.07	0.22
Perceived threat × social identity	0.03	−0.03	0.08	0.02*^a^*	0.00	0.05	0.01	−0.02	0.04
*Threat effect for low identifiers*	−0.08***	−*0.12*	−*0.04*	−0.09***	−*0.12*	−*0.05*	−0.07***	−*0.11*	−*0.04*
*Threat effect for high identifiers*	−0.06***	−*0.09*	−*0.04*	−0.05***	−*0.08*	−*0.02*	−0.06***	−*0.09*	−*0.03*
**Outcome: well-being**									
Self-esteem	0.82***	0.74	0.90	0.82***	0.74	0.90	0.82***	0.74	0.91
Perceived threat	−0.07***	−0.11	−0.03	−0.07**	−0.11	−0.03	−0.07**	−0.11	−0.03
Social identity	0.06	−0.11	0.22	0.17***	0.08	0.27	0.04	−0.09	0.16
Perceived threat × social identity	0.03	−0.06	0.13	0.01	−0.04	0.05	0.00	−0.05	0.06
*Threat effect for low identifiers*	−0.09**	−*0.16*	−*0.02*	−0.07**	−*0.13*	−*0.02*	−0.07**	−*0.13*	−*0.01*
*Threat effect for high identifiers*	−0.06**	−*0.11*	−*0.02*	−0.07**	−*0.11*	−*0.02*	−0.07**	−*0.11*	−*0.02*

^a^p < 0.10; *p < 0.05; **p < 0.01; ***p < 0.001. LL, lower limit; UL, upper limit. The italics are added to indicate slope effects.

Second, self-esteem further predicted well-being, and while perceived threat again negatively predicted well-being in each model, only Gulf identity was positively related to well-being. None of the interaction terms reached conventional levels of significance, and simple slope effects corroborated this by showing similarly negative effects of threat among low vs. high identifiers.

Table 3 further delineates the total, direct, and indirect effects of perceived threat on well-being, at different levels of social identification. The results in each of these models revealed significant total and direct effects of threat on well-being for those with low, medium, and high levels of social identification alike. Importantly, the significant indirect effects point to self-esteem as mediator of the threat-well-being relationship. This mediating effect was significant at all levels of (each facet of) identity.

**TABLE 3:** Unstandardized estimates and 95% confidence intervals of the total, direct, and indirect, effects of perceived threat on well-being *via* self-esteem, at different levels of each facet of social identity separately.

	Total			Direct			Indirect		
	Estimate	95% CI		Estimate	95% CI		Estimate	95% CI	
	b	LL	UL	b	LL	UL	b	LL	UL
*Moderator: Qatar identity*									
Low identity	−0.15***	−0.23	−0.08	−0.09**	−0.16	−0.02	−0.07**	−0.11	−0.02
Medium identity	−0.12***	−0.17	−0.08	−0.07***	−0.11	−0.03	−0.05**	−0.08	−0.03
High identity	−0.11***	−0.16	−0.07	−0.06**	−0.11	−0.02	−0.05**	−0.08	−0.03
*Moderator: gulf identity*									
Low identity	−0.14***	−0.21	−0.08	−0.07**	−0.13	−0.02	−0.07**	−0.10	−0.04
Medium identity	−0.12***	−0.17	−0.08	−0.07**	−0.11	−0.03	−0.05**	−0.08	−0.03
High identity	−0.11***	−0.16	−0.05	−0.07**	−0.11	−0.02	−0.04**	−0.07	−0.02
*Moderator: Arab identity*									
Low identity	−0.13***	−0.20	−0.07	−0.07**	−0.13	−0.01	−0.06**	−0.10	−0.02
Medium identity	−0.12***	−0.17	−0.08	−0.07**	−0.11	−0.03	−0.05**	−0.08	−0.03
High identity	−0.12***	−0.17	−0.06	−0.07**	−0.11	−0.02	−0.05**	−0.08	−0.02

^a^p < 0.10; *p < 0.05; **p < 0.01; ***p < 0.001. LL, lower limit; UL, upper limit.

Finally, as an additional robustness check, we created indices of comparative identity (see Huici et al., 1997). Particularly, we produced variables that compared each facet or “level” of identity with the other two and reran the analyses using these comparative identity scores as moderators. The results are portrayed in Table 4 and, interestingly, seem to mirror the original results, as such providing more confidence in our conclusions. Specifically, identifying relatively more as Qatari compared to as Khaleeji or Arab did not moderate the association between threat and self-esteem, nor did identifying relatively more as Arab compared to as Khaleeji or Qatari. The only significant moderator was identifying relatively more as Khaleeji compared to as Qatari or Arab. Simple slope analyses indicated that this comparative Gulf identity buffered the negative threat effects, in the sense that the threat effect was considerably lower for those who most strongly identified with the Gulf area compared with their level of identification with Qatar or with the whole Arab world. Put differently, this comparative supranational, regional identity aspect of identifying relatively most with the Gulf area significantly weakened the psychological effects of the threat following the blockade, whereas the other two comparative identities (either at the lower, national or the higher, religious level) did not exert such moderating effects. In sum, the saliency of the Gulf identity mattered most.

**TABLE 4:** Unstandardized estimates and 95% confidence intervals of moderated mediation analyses on self-esteem (upper panel) and well-being (lower panel) for each facet of comparative social identity separately.

	Moderator:								
	Comparative Qatar identity			Comparative Gulf identity			Comparative Arab identity		
	Estimate	95% CI		Estimate	95% CI		Estimate	95% CI	
Outcome: self-esteem	b	LL	UL	b	LL	UL	b	LL	UL
Perceived threat	−0.06***	−0.09	−0.04	−0.07***	−0.09	−0.04	−0.07***	−0.09	−0.04
Social identity	−0.17***	−0.23	−0.11	0.10***	0.05	0.16	0.04	−0.04	0.11
Perceived threat × social identity	−0.03	−0.07	01	0.03*	0.00	0.07	−0.02	−0.06	0.03
*Threat effect for low identifiers*	−0.05**	−*0.08*	−*0.01*	−0.09***	−*0.12*	−*0.06*	−0.06***	−*0.09*	−*0.03*
*Threat effect for high identifiers*	−0.08***	−*0.11*	−*0.05*	−0.04*	−*0.08*	−*0.01*	−0.08***	−*0.11*	−*0.04*
**Outcome: well-being**									
Self-esteem	0.87***	0.79	0.95	0.88***	0.80	0.96	0.82***	0.74	0.91
Perceived threat	−0.06**	−0.11	−0.02	−0.07**	−0.11	−0.02	−0.07**	−0.11	−0.02
Social identity	−0.21***	−0.31	−0.10	0.19***	0.10	0.28	−0.06	−0.18	0.06
Perceived threat × social identity	0.00	−0.06	0.06	0.00	−0.05	0.06	−0.01	−0.08	0.06
*Threat effect for low identifiers*	−0.06**	−*0.12*	−*0.01*	−0.07**	−*0.12*	−*0.01*	−0.06**	−*0.12*	−*0.01*
*Threat effect for high identifiers*	−0.06**	−*0.12*	−*0.01*	−0.06**	−*0.12*	−*0.01*	−0.07**	−*0.13*	−*0.01*

^a^p < 0.10; *p < 0.05; **p < 0.01; ***p < 0.001. L, lower limit; UL, upper limit. The italics are added to indicate slope effects.

## Discussion

The aim of the current study was to investigate the additive and interactive effects of threat and social identity in predicting self-esteem and well-being among Qatari youth. Indeed, it can be argued that the 2017 Qatar blockade may have increased feelings of threat, particularly among this group, and whereas such threat perceptions might negatively impact well-being *via* lowered self-esteem, we predicted that an overarching social identity might mitigate (i.e., buffer) this negative association. Our results somewhat provided support for this hypothesis. Indeed, the overarching supra-regional identity facet of Gulf (Khaleeji) identity not only positively predicted self-esteem and well-being, it also buffered the negative threat-self-esteem association. Specifically, among those identifying more strongly with the Gulf region, the threat perceptions accompanying the blockade did not relate to lower self-esteem to the same extent (as opposed to among low Gulf identifiers). Interestingly, both identification with the “lower” national (Qatar) identity facet and with the “higher,” more encompassing ethno-cultural/religious (Arab) identity facet failed to exert such buffering effect.

### Applying Intergroup Threat and Social Identity Theory to Intergroup Relations in the Gulf Region

It has been argued that social identity theory has not been as impactful as expected in political psychology due to a focus on the effects of social identity across group memberships in lab contexts (within Western societies), and a lack of attention on the development of such identities in real-world contexts (outside the labs of Western universities, see Huddy, 2001). It is important to note the challenges to social identity theory, as outlined by Huddy (2001), also reflect some of the original theorizing by Tajfel et al. (1971), who emphasized the importance of understanding the role of context when applying the theory to different groups and to not make the assumption of universality. Nonetheless, a recent cross-cultural meta-analysis including over 20,000 respondents across 18 studies/samples found ingroup bias to be a relatively universal phenomenon (Fischer and Derham, 2016), but with systematic variance found for countries with differing levels of individualism or collectivism, and uncertainty avoidant contexts. Importantly, and reflecting limitations found in much of the literature, no data on Muslim-majority countries were included, thereby limiting the generalizability of the findings.

Relatedly, while there is extensive research on the effects of political violence (Palmieri et al., 2008; Muldoon, 2013), less is known about the complex relationship between the perception of threat in socio-political tensions, and the potential buffering effect of identity in its associations with mental health variables such as self-esteem. This is especially the case for younger populations. In fact, because the blockade of Qatar is experienced as an act of aggression, it can be seen like a perceived threat to social identity and national security. In those circumstances, people may perceive this threat as a source of anxiety and worry. But when the crisis is well managed, a collective consciousness can develop protective attitudes and resilient actions to overcome the threat by reinvesting and re-constructing their social identity and collective social esteem.

Threats to the content and value of group membership are distinguished in social identity theory (Breakwell, 1983; McKeown et al., 2016). When individuals’ social identity is threatened, they attempt to raise their esteem. Worchel and Coutant (1997) maintain that threats to our identities may come from out-groups: when such out-groups attack our in-group, this threat can severely worsen our self-worth and well-being. Studies showed that perceived threat to our group identities leads to intergroup anxiety, and but very few attempted to examine the potential buffering role the identities we attempt to portray may play (Branscombe et al., 1999). An exception can be found in the work of Chen et al. (2015). In a South African and a Chinese sample, they found strong effects of (environmental and financial) threat on lower well-being and higher ill-being, and although relatedness (a construct closely related to group identification) was positively related to well-being, it failed to buffer against the detrimental threat effects—put differently, they also found additive but no interactive effects.

### What Do We Know About the Psychology of Qataris?

Compared to studies involving Western samples, there is a lack of high-quality large-scale datasets concerning populations from Gulf States such as Qatar. Within psychology, and social sciences more broadly, the dependence on samples typically from WEIRD populations has limited the cross-cultural generalizability of key conceptualizations of the self, motivation, and behavior (Henrich et al., 2010). Nonetheless, there has been an emergence of some empirical research on youth well-being from Gulf countries in international psychology literature (Abdel-Khalek, 2011, 2013; Al-Attiyah and Nasser, 2016; Bedair et al., 2020).

The potential role of the Qatari blockade and its influence on youth national, regional, and ethno-cultural identity, self-esteem, and well-being presents a dilemma for political psychology conceptualizations of intergroup conflict. The combined effects of these variables and their potential association with well-being in Qatari youth is poorly understood. This paper addressed this issue, and its results s present a number of interesting findings in the context of the unique challenges brought about by the Qatari blockade for Qatari youth. Firstly, it is clear that despite these challenges, young Qataris perceived relatively low levels of threat, and scored relatively high on all facets of identity, self-esteem, and well-being. In line with our predictions, those who perceived lower levels of threat and had higher levels of identification and self-esteem were more likely to report stronger levels of overall well-being. In contrast to prior literature suggesting that social identification increases due to perceived threat (Haslam et al., 2005), this model provides no evidence that this was directly the case within the Qatari blockade.

Most importantly, and partly confirming our predictions based on a firm theoretical background, this study also found a (marginally significant) buffering effect of superordinate identification with the Gulf region as a whole, but not with national or Arab identification. This may be due to the relatively untested psychometric properties of the measure used, but it may also be attributed to the fact that young Qataris already had high levels of national identity prior to the blockade. Given the absence of valid and reliable representative data for the mental health of Qatari youth prior to the blockade, it is not possible to infer direct causal effects of the blockade at present. Nonetheless, we put forward that future studies could further explore the roles of different “levels” of identification. Although all facets of identity positively relate to positive outcomes such as self-esteem and well-being, only a “medium-level” identity can (to a limited extent) buffer against negative effects of intergroup threat perceptions.

### Khaleeji Identity as a “Cure for All”?

On the one hand, a national identity could be too narrow to offer solace after an impactful event (such as the blockade) that heightens intergroup tensions in the area. As the blockade might have especially triggered those strongly identifying with their country, one could have even expected an opposite interaction effect here, with strong national identification amplifying the threat effects. On the other hand, the superordinate identification with all Arabs globally might be too broad to buffer threat effects either. Indeed, the blockade worsened intergroup relations in the Gulf area only. As a consequence, a strong ethno-cultural identity would not be powerful enough to limit the effects of threat on self-esteem and well-being. It seems that the overarching identity with the Gulf area (i.e., the perpetrator of the blockade) is just about right to buffer against the potential detrimental effects of the blockade. And even this facet of Khaleeji identity has its limits, as the threat effect remained significant among high Gulf identifiers.

In the beginning of the 21st century, the Arab Gulf countries entered a new era, an era during which it drew its power, not only through its oil production, but also through possessing cultural capacities (Alsharekh and Springborg, 2012). The Gulf countries have succeeded in shaping a Gulf identity, as they considered doing that was necessary to maintain national, regional, and international legitimacy (Allam and Karolak, 2020). This identity used to provide the citizens of those countries with a sense of safety, trust, and positive self-image. Historically, the Gulf identity emerged with the establishment of the Gulf Cooperation Council in 1981. The purpose of this council was presenting a unified vision of the Arab Gulf regarding external political affairs which reflect one Gulf identity.

The Gulf Cooperation Council members worked hard to manifest this idea to a living reality (Lawson, 2012), and they succeeded to a certain extent in doing so. These countries have strong social, familial relations, and homogenous patterns in sports and cultural expression’such as poetry and music that they share. This homogeneity is what makes Gulf countries standout from the rest of Arab countries. The Gulf countries participated in huge projects for rewriting their mutual history, traditions, sports and arts (Erskine-Loftus et al., 2016).

However, the recent Gulf crisis and the blockade of Qatar demonstrated the fragility of this political homogeneity amongst them. This crisis has also impacted aspects of identity for Gulf countries; especially for Qataris, since the blockade, the threats, and the media conflicts -especially on social media- resulted in an identity wound, where the Gulf identity was thought to have lost its value and importance as a source of safety and trust. There was much anecdotal reports and media coverage regarding the Qatari resilience in response to the blockade, and it remains unknown why the specific facet of Khaleeji identity provided a path to such resilience. It may be related to the web of identities fused between Qatari citizens and their fellow GCC countries, and the potentially complex depths of the Khaleeji identity (which itself predates the national GCC identities; Al-Misned, 2016; Allam and Karolak, 2020). As such, we claim that the Gulf region is characterized by distinct ingroup-outgroup dynamics, which in itself form an interesting avenue for future work. For instance, experimental co-ethnic voting evidence from Qatar indicates that Qataris have low political salience of ethnic divisions, and do not exhibit negative prejudice against perceived outgroup members (Shockley and Gengler, 2020). Perhaps such low outgroup negativity is the exact reason why a strong Gulf identity is maintained, even in the face of the blockade.

Taken together, these nuanced and interlinking identity features could impact the identification of cause-and-effect relationships between the blockade and different levels of identity, mental health, and well-being. The interconnected nature of Qatari identity within the GCC means that the shared descent of all stakeholders could be separable from the perceived threat of the blockade with respect to other factors (i.e., economic problems). It is also possible that Qatari youth were able to set apart their shared identity with the blockading GCC countries from the socio-political conflicts underpinning the blockade.

### Implications for Education in Qatar

The Qatari National Human Rights Committee (QNHRC) in 2017 very clearly highlighted the adverse impact of the blockade on all aspects of society, including youth mental health. Coupled with a comparatively high prevalence of mental health problems (Schoenbach et al., 2018; Khaled, 2019), in addition to the enduring role of stigma in the region (Ciftci et al., 2013; Zolezzi et al., 2017), there are several educational lessons from which to strengthen societal understanding of the connections between Qatari youth identity, perceived threats in the present or future, self-esteem, and well-being. Given the salient effects of threat and uncertainty on youth mental health, awareness-raising campaigns and the psychoeducation of how humans react to (perceived) threat can assist in helping young Qataris understand their reaction to the blockade, and indeed any future changes. Further initiatives that can solidify and support youth Khaleeji identity in the face of (perceived) threats of the blockade are likely to positively impact outcomes for Qatari youth, but more formal evaluations of such interventions are nonetheless advisable. Indeed, in order to offset or minimize any potentially adverse mental health consequences, educational psychologists in Qatar may be appropriately placed to facilitate new awareness and psychoeducational campaigns to help young Qataris understand the functionality of identity in all its facets and all its complexity, especially in the face of perceived threats and uncertainty.

### Strengths and Limitations

Despite the fact that we used a very large sample from a non-WEIRD country, in a unique context to perform this integrative test of three well-known predictors of well-being, there are a number of methodological considerations in this study. It is important to note that the time of data collection for this study was in mid-2020. Owing to the considerable gap in time between the initial shock of the blockade and the 2020 pandemic, it is conceivable that this time lag effect may have impacted the quality of the data. Given the developmental changes of the young Qatari population from the time of the blockade up until this study’s data collection, it is likely that respondents could have had different responses during the blockade. It is also possible that Qatari youth consolidated and enhanced their national sense of identity within this time period, but this was not possible to retrospectively demonstrate within the methodology of this study. Furthermore, given the nature of the research questions, it would have been useful to compare data before and after the blockade; but no such data presently exists for the population of interest. Moreover, the psychometric properties of the national identity measure and also the perceived threat require further validation in Gulf populations before concrete interpretations can occur. Especially our measure of threat stands rather far from classic threat scale that tap into a certain symbolic or realistic feeling of threat with outgroup members (mostly newcomers) “taking away” part of the ingroup culture or ingroup socio-economic status. Our measure contains more anxiety-related items referring to the blockade, as such tapping into threat coming from outgroups that are not present within the own country, but still pose a major concern (particularly in terms of economic threat). A final limitation to be considered is the role of socially desirable responses when interpreting the overall dataset.

## Conclusion

In the context of intergroup relations within the Gulf area, we showed that perceived threat accompanying the 2017 Qatar blockade was related to Qatari youngsters’ lower well-being *via* lower levels of self-esteem. Conversely, identification with the nation of Qatar, the Gulf area, and the Arab world as a whole were all strongly positively associated with self-esteem and well-being, and the specific facet of Gulf (i.e., Khaleeji) identity even buffered against some detrimental threat effects. Given the new forms of intergroup conflict that are evolving—such as cyberwarfare, or sanctions and restrictions due to pandemic and/or geopolitical factors—researchers should examine the real-time development of perceived threat among different age cohorts, in addition to embedding other variables that can capture the potential role of individual differences. Additionally, future research should endeavor to collect representative samples from across the Gulf region to ensure greater comparability across the collective literature. This will aid the advancement of a more globally representative political psychology where the GCC region can be appropriately situated.

## Data Availability Statement

The raw data supporting the conclusions of this article will be made available by the authors, without undue reservation.

## Ethics Statement

The studies involving human participants were reviewed and approved by the IRB and was obtained from the Doha Institute for Graduate Studies. The patients/participants provided their written informed consent to participate in this study.

## Author Contributions

AA reviewed the analyzed data and supported the writing of the manuscript. JV and DM performed data analysis and supported the drafting and editing of the manuscript. JV, AA, and MA analyzed the data and drafted the manuscript. DA-A and MA conducted the data collection. JV and YH reviewed the analysis and supported the writing of the manuscript. All authors read and approved the final manuscript.

## Author Disclaimer

The findings herein reflect the work, and are solely the responsibility, of the authors.

## Conflict of Interest

The authors declare that the research was conducted in the absence of any commercial or financial relationships that could be construed as a potential conflict of interest.

## Publisher’s Note

All claims expressed in this article are solely those of the authors and do not necessarily represent those of their affiliated organizations, or those of the publisher, the editors and the reviewers. Any product that may be evaluated in this article, or claim that may be made by its manufacturer, is not guaranteed or endorsed by the publisher.

## Footnotes

1 We performed additional analyses that also included gender at the school level in the model, but there were no differences in self-esteem and well-being between boys- and girls-schools (*b* = 0.04, *p* = 0.43 for self-esteem; *b* = −0.05, *p* = 0.59 for well-being), nor were there cross-level interaction effects between individual threat and school-level gender (*b* = 0.00, *p* = 0.93 for self-esteem; *b* = 0.01, *p* = 0.77 for well-being).
